# Identification and Characterisation of a Novel Acylpeptide Hydrolase from *Sulfolobus Solfataricus*: Structural and Functional Insights

**DOI:** 10.1371/journal.pone.0037921

**Published:** 2012-05-24

**Authors:** Marta Gogliettino, Marco Balestrieri, Ennio Cocca, Sabrina Mucerino, Mose Rossi, Mauro Petrillo, Emanuela Mazzella, Gianna Palmieri

**Affiliations:** 1 Institute of Protein Biochemistry, National Research Council (CNR-IBP), Naples, Italy; 2 CEINGE - Biotecnologie Avanzate, Via Gaetano Salvatore, Naples, Italy; University of Minho, Portugal

## Abstract

A novel acylpeptide hydrolase, named APEH-3*_Ss_*, was isolated from the hypertermophilic archaeon *Sulfolobus solfataricus*. APEH is a member of the prolyl oligopeptidase family which catalyzes the removal of acetylated amino acid residues from the N terminus of oligopeptides. The purified enzyme shows a homotrimeric structure, unique among the associate partners of the APEH cluster and, in contrast to the archaeal APEHs which show both exo/endo peptidase activities, it appears to be a “true” aminopeptidase as exemplified by its mammalian counterparts, with which it shares a similar substrate specificity. Furthermore, a comparative study on the regulation of *apeh* gene expression, revealed a significant but divergent alteration in the expression pattern of *apeh-3_Ss_* and *apeh_Ss_* (the gene encoding the previously identified APEH*_Ss_* from *S. solfataricus*), which is induced in response to various stressful growth conditions. Hence, both APEH enzymes can be defined as stress-regulated proteins which play a complementary role in enabling the survival of *S. solfataricus* cells under different conditions. These results provide new structural and functional insights into *S. solfataricus* APEH, offering a possible explanation for the multiplicity of this enzyme in Archaea.

## Introduction

Acylpeptide hydrolase (APEH), also referred to as acylaminoacyl-peptidase, is a member of the prolyl oligopeptidase (POP) family of serine peptidases (clan SC, family S9) [1], [2]. It catalyzes the removal of N-terminally blocked amino acid residues from peptides, producing an acetyl amino acid and a peptide with a free N terminus shortened by one amino acid residue [2], [3]. The enzyme cleaves a variety of peptides with different N-terminal acyl groups, such as acetyl, chloroacetyl, formyl and carbamyl [2].

To date, these enzymes have been studied in a number of eukaryal organisms [4], [5] and in some Archaea [6]–[9] but never in Bacteria. Rat [10], porcine [11], human [12] and bovine [4], [5] APEHs from different tissues, including blood, brain and liver, show significant sequence identity and are reported to form homotetramers. The role of this enzyme in the physiology of the cell is not clear; however, it has been recently suggested that it is involved in the protein-degradation machinery [13] and can function as a secondary antioxidant defense system for proteins damaged by oxidative stress [14]–[17]. APEH may therefore represent a promising therapeutic target relevant for a wide array of diseases caused by misfolded protein accumulation, associated, for example, with the development and progression of cancer [18]–[20].

Among the APEH enzymes, only one three-dimensional structure, that of the APEH from *Aeropyrum pernix* K1, coded by the gene *ape1547.1* (APEH*_Ap1547.1_*), has been reported [6], [21]. Unlike the tetrameric mammalian APEHs, APEH*_Ap1547.1_* is a symmetric homodimer with each subunit containing two domains: the N-terminal β-propeller, and the peptidase domain with an α/β-hydrolase fold, characteristic of this enzyme family [1], [22]. The catalytic triad of APEH*_Ap1547.1_* consists of Ser445, Asp524 and His556 and the hydrophobic S1 substrate binding pocket accepts large non-polar residues, such as phenylalanine and leucine. This binding site is different from that of the porcine enzyme [11], which is specific for the small alanine side-chain. Moreover, kinetic analysis using different peptide substrates revealed that APEH*_Ap1547.1_*, which was regarded as an exopeptidase, also exhibited endopeptidase activity [6]. Two further archaeal APEHs have been so far isolated and characterized from *Pyrococcus horikoshii* coded by the genes *ph0863* (APEH*_Ph0863_*) [7] and *ph0594* (APEH*_Ph0594_*) [8]. Compared to their mammalian counterparts, these archaeal enzymes are between 90–150 residues shorter, and their molecular, structural and functional properties are quite similar, but the hexameric architecture of the APEH*_Ph0594_* [8] is unique among both known APEHs and the members of the POP family.

We recently described the identification and characterization of a new acylpeptide hydrolase, named APEH*_Ss_* (coded by the gene *sso2141*) from the hyperthermophile *Sulfolobus solfataricus* [9]. The enzyme is a cytosolic homodimeric protein, displaying exopeptidase and endopeptidase activities similar to the homologous APEHs from *Aeropyrum pernix* and *Pyrococcus horikoshii*. Moreover, the 3D model of APEH*_Ss_*, constructed using the crystal structure of APEH*_Ap1547.1_* as a template, showed the typical structural features of the POP family including an N-terminal β-propeller and a C-terminal α/β hydrolase domain.

In the present study we have isolated and characterized a novel archaeal member of the APEH family from *S. solfataricus* coded by the gene *sso2693* and named APEH-3*_Ss_*. The protein is a cytosolic exopeptidase possessing *pseudo*-endopeptidase activity, consisting of 591 amino acids and sharing 15% sequence identity with the previously identified APEH*_Ss_*. Interestingly, our investigations on the isolated enzyme show the presence of intermediate features between those from archaeal and mammalian APEHs. Moreover, while APEH*_Ss_* appeared to be largely distributed in several related archaeal species, putative APEH-3*_Ss_*-homologs were found only in *Crenarchaeota phylum*. Finally, study on the regulation of gene expression revealed that both *apeh_Ss_* and *apeh-3_Ss_* genes showed distinct expression patterns in response to different stressful growth conditions, suggesting that the two enzymes can be described as stress-regulated proteins playing a complementary role in enabling the survival of *S. solfataricus* cells under different conditions.

## Results and Discussion

### Purification of a novel acylpeptide hydrolase from *Sulfolobus solfataricus*

Although the structure-function relationship of acylpeptide hydrolases (APEH) in Archaea has been studied extensively [6]–[9], [21], [23] relatively little is known about the biological function of these proteins. The sequenced genome of *S. solfataricus* P2 revealed the presence of three ORFs homologues to APEH such as *sso1419*, *sso2141* and *sso2693*. Recently, the *sso2141* gene product, namely APEH*_Ss_* (Uniprot accession number Q7LX61), has been characterized [9]. It is a cytosolic homodimer protein with a molecular mass of 125 kDa displaying similar properties to the homologous APEH enzymes isolated from *A. pernix* (Uniprot accession number Q9YBQ2, APEH*_Ap1547.1_*) [6], [21] and *P. horikoshii* (Uniprot accession numbers O5832 and O58593 for APEH*_Ph0594_* and APEH*_Ph0863_*, respectively) [7], [8]. Interestingly, unlike the mammalian members of the APEH family, archaeal APEHs appear to be present in multiple isoforms corresponding to a variety of *apeh* genes, but whether this multiplicity corresponds to distinct biological functions is still unclear. Therefore, to gain insight into the physiological roles of these archaeal APEH enzymes, as a first approach, we decided to investigate the kinetic and structural features of new APEHs from *S. solfataricus*, in order to compare their properties with those of the previously characterized enzymes from archaeal sources [9].

**Figure 1:**
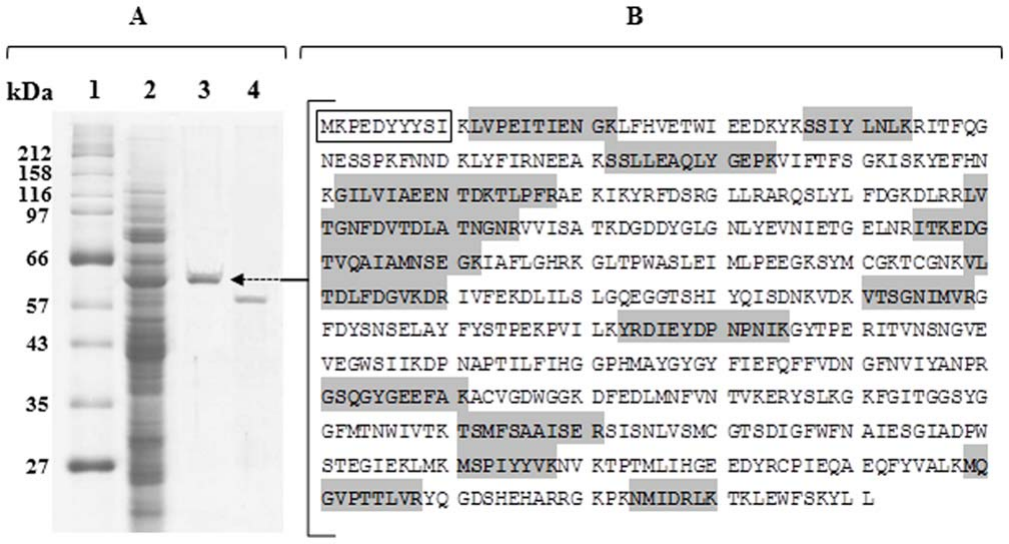
Purification and sequence analysis of APEH-3*_Ss_* from *Sulfolobus solfataricus*. (**A**) SDS-PAGE of purified APEH-3*_Ss_*; lane 1, molecular weight markers (myosin 212.0 kDa, MBP-β-galactosidase 158.0 kDa, β-galactosidase 116.0 kDa, phosphorylase b 97.2 kDa, serum albumin 66.4 kDa, glutamic dehydrogenase 56.6 kDa, MBP2 *E. coli* 42.7 kDa, thioredoxin reductase 34.6 kDa and triosephosphate isomerase 27.0 kDa); lane 2, protein pattern of the cytoplasmic fraction obtained after cell lysis; lane 3, purified APEH-3*_Ss_*; lane 4, purified APEH*_Ss_*. (**B**) Amino acid sequence deduced from the APEH-3*_Ss_*-coding gene (*sso2693*). The peptides identified by LC-MS/MS are highlighted in grey and the N-terminal amino acid sequence of the purified protease is boxed.

In setting up the purification procedure, the choice of the first chromatographic step was critical to obtain effective separation of the isoenzymes, using N-acetyl-leucine-para-nitroanilide (Ac-L-*p*NA) as a substrate. By way of a combination of hydrophobic affinity and anion exchange chromatography, a new acylpeptide hydrolase (named APEH-3*_Ss_*, uniprot accession number Q97VD6) was isolated. The protein was purified about 6800-fold, with an activity recovery of about 27% and a specific activity of 4800 mU mg^−1^ as summarized in **Table 1**. The homogeneity of the final product was determined by SDS-PAGE (**Fig. 1A**), mass spectrometry and N-terminal sequence analyses.

**Table 1:** Purification procedure of APEH-3*_Ss_* enzyme from *S. solfataricus*.

Purification step	Total protein (mg)	Total activity ?>(mU)*	Specific activity (mU mg^−1^)	Yield (%)	Purification fold
**Cellular extract**	279	181	0.7	100	1
**Q Sepharose**					
**APEH-3** ***_Ss_***	20.3	122.4	6.0	67.6	8.6
**APEH** ***_Ss_***	33.0	53.6	1.6		
**Phenyl Sepharose**	1.5×10^−1^	91.2	608	50.4	869
**Mono Q**	0.8×10^−1^	84.0	1050	46.4	1500
**Mono S**	0.1×10^−1^	48.0	4800	26.5	6857

* One unit of APEH activity (U) is defined as the amount of enzyme required to hydrolyze 1 µmol of Ac-L-*p*NA per min under the assay conditions.

It is worth noting that during the purification procedure, apart from the already characterized APEH*_Ss_*, no further acylpeptide hydrolase activity was detected, even when using different acetyl substrates, suggesting that our culture conditions were unfavorable for expression of all putative APEH isoenzymes from *S. solfataricus*.

### Identification of the purified protein

The purified protease (APEH-3*_Ss_*) was subjected to *in-gel* digestion and the tryptic peptides were analyzed by nano-HPLC-ESI-MS/MS. The product of *sso2693* gene of *S. solfataricus P2* was identified with 30% sequence coverage (**Fig. 1B**, **Table S1**) and no significant protein contaminants were retrieved. Moreover, Edman degradation analysis of APEH-3*_Ss_* revealed a unique N-terminal sequence MKPEDYYYSI (**Fig. 1B**) with the first amino acid corresponding to the N terminus of the polypeptide translated from the *sso2693* gene, suggesting that the protein is not processed and confirming its cytosolic localization. APEH-3*_Ss_* is a new *S. solfataricus* member of the APEH family, consisting of 591 amino acids (**Fig. 1B**); unexpectedly, sequence similarity searches revealed a low identity between APEH-3*_Ss_* and the homologous APEH*_Ss_* from *S. solfataricus* (15% sequence identity) [9], although this value increased to 28% when analyzing the catalytic domain in the C-terminal region, which contains several clusters of identical residues. In this region the highest sequence identity (44%) was found between APEH-3*_Ss_* and the APEH from the hyperthermophile *P. horikoshii* (APEH*_PH0863_*) [7], whilst APEH*_Ap1547.1_* [6], [21] and APEH*_Ph0594_* [8] shared 23% and 40% identity with APEH-3*_Ss_*, respectively.

In addition, APEH-3*_Ss_* showed significant similarity (about 25% identity) with mammalian APEHs and comparable values were obtained with several mammalian APEHs, suggesting a structural relationship between APEH-3*_Ss_* and these eukaryal enzymes (**Table S2**). Multiple sequence alignment of the C-terminal region of APEHs from different sources is shown in **Figure S1**. The typical structural features of the POP family are conserved in APEH-3*_Ss_*, such as the catalytic triad (S, D, H) and the consensus sequence G-X-S-X-G-G, including the active site serine residue and the three glycine which improve the binding of substrate by preventing steric hindrance. Moreover, the alignment revealed the presence of a conserved motif of four amino acids (HGGP), which comprises the glycines of the oxyanion hole [24]. Unfortunately, the low sequence identity between APEH-3*_Ss_* and APEH*_Ap1547.1_* did not allow the construction of a 3D model for APEH-3*_Ss_* using the only available crystal structure of APEH*_Ap1547.1_* as a template.

### Molecular properties of APEH-3*_Ss_*

The molecular mass of the purified protein (64 kDa), as determined by SDS-PAGE analysis (**Fig. 1A**), was consistent with that calculated on the basis of the amino acid sequence (67 kDa) (**Fig. 1B**) deduced from the coding *sso2693* gene. The degree of oligomerization of APEH-3*_Ss_* was investigated by gel filtration chromatography using two different size-exclusion columns. These analyses indicated that APEH-3*_Ss_* is a trimeric enzyme with an apparent molecular mass of 192 kDa, composed of identical monomers of 64 kDa. Among all of the characterized APEHs from archaeal or mammalian sources, this structure represents a unique oligomerization state, suggesting that APEH-3*_Ss_* could be involved in different biological processes.

**Figure 2:**
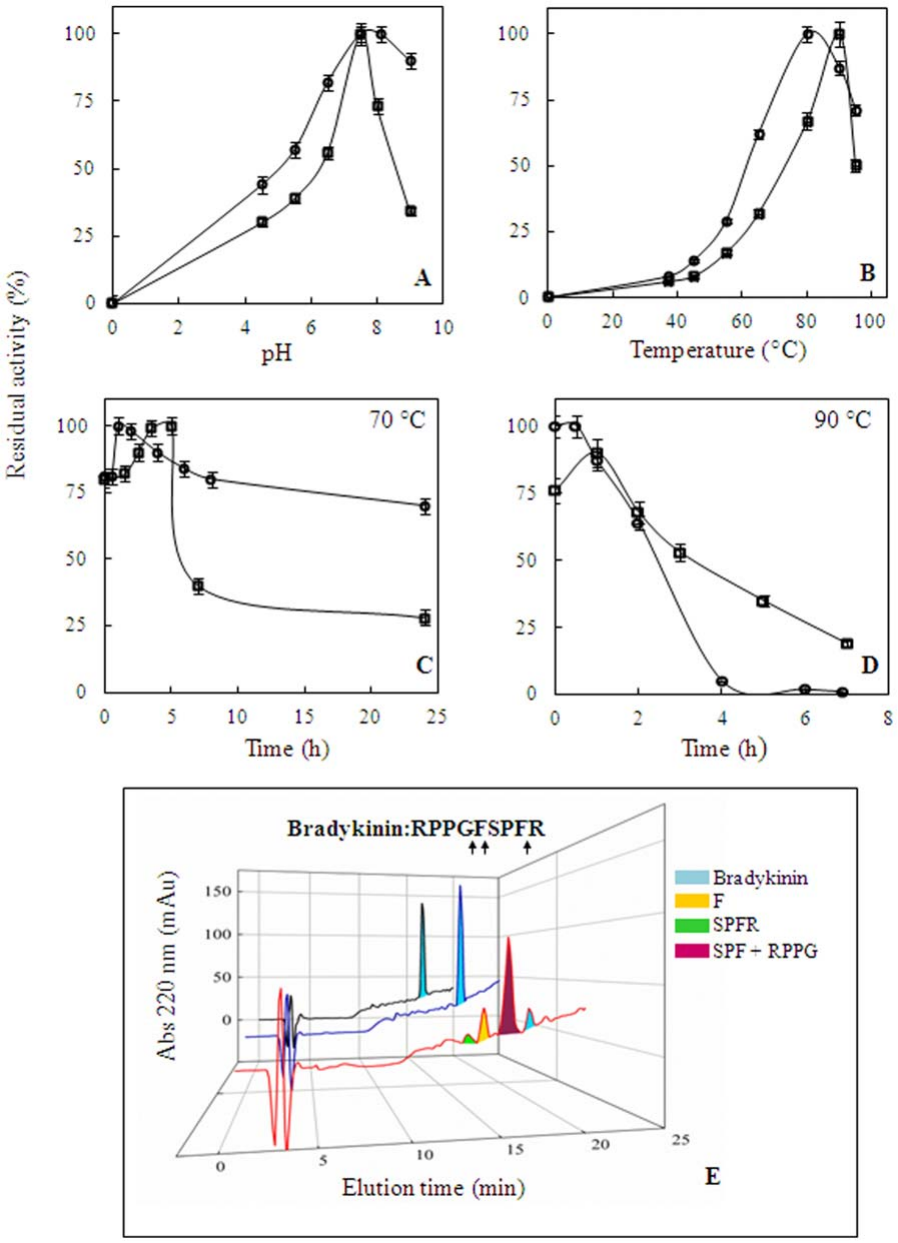
Functional and molecular properties comparison of APEH-3*_Ss_* and APEH*_Ss_*. (**A**) pH-activity profile of APEH*_Ss_* and APEH-3*_Ss_*. (**B**) Influence of temperature on the enzyme activities. (**C–D**) Thermoresistance of both APEH detected at (**C**) 70°C and (**D**) 90°C. In all pictures APEH*_Ss_* and APEH-3*_Ss_* are indicated by a circle (○) or square (□) respectively. (**E**) RP-HPLC analysis of proteolytic digests of bradykinin with APEH*_Ss_* (**^___^**) or APEH-3*_Ss_* (**^___^**). Control sample (**^___^**) was run in parallel in the absence of the enzymes and the chromatogram traces are shown. Colored peaks isolated after 30 min of incubation were collected and the identification of the peptides generated was performed by Edman degradation analysis. The enzyme cleavage specificity, revealed by the peptide fragments assigned along the bradykinin sequence, is indicated by arrows.

In evaluating the molecular properties of the two *S. solfataricus* APEH enzymes, we found that APEH-3*_Ss_* showed a bell-shaped pH-activity profile with an optimal pH value of 7.5 (**Fig. 2A**), in strong agreement with that reported for APEH*_Ss_* [9], using Ac-L-*p*NA as substrate. However, above pH 7.5, APEH-3*_Ss_* activity drastically decreased compared to that of APEH*_Ss_*, possibly due to the presence of a different electrostatic environment around the active site, which changes progressively with the pH increase, thus impairing the catalytic machinery. The temperature-activity profile (**Fig. 2B**) of APEH*_Ss_* showed a broad range (from 60 to 90°C) in comparison to that observed for APEH-3*_Ss_*. The optimal temperatures were 90°C and 80°C for APEH-3*_Ss_* and APEH*_Ss_*, respectively, and a high sensitivity to temperature was observed for APEH-3*_Ss_* activity which rapidly decreased over 90°C (**Fig. 2B**). Moreover, by analyzing the thermo-resistance of both APEHs (**Fig. 2C, D**), it was observed that APEH-3*_Ss_* retained 30% activity against 75% determined for APEH*_Ss_* [9], following the incubation for 24 h at 70°C, while the residual activity of APEH-3*_Ss_* was halved after 4 h incubation at 90°C which instead produced complete inactivation of APEH*_Ss_*. In addition APEH-3*_Ss_* appeared to slightly increase its relative activity during the first 5 hours of incubation (70°C), sharply declining thereafter. This behavior may suggest a possible conformational change by heat treatment producing a transient positive effect on enzyme activity as somehow observed for the APEH from *P. horikoshii* [7], [8].

### Endopeptidase and exopeptidase activity of APEH-3*_Ss_*

In order to better understand the physiological role of APEH-3*_Ss_* we conducted a comparative kinetic study of APEH-3*_Ss_* and previously characterized APEHs from different sources. Under optimal assay conditions, APEH-3*_Ss_* exhibited hydrolytic activity towards Ac-L-*p*NA, N-acetyl-alanine-para-nitroanilide (Ac-A-*p*NA) and N-acetyl-phenylalanine-para-nitroanilide (Ac-F-*p*NA) but not *versus* L-*p*NA, A-*p*NA or F-*p*NA as substrates, confirming that APEH-3*_Ss_* is a true acylpeptide hydrolase. Interestingly, no significant variation was observed in the binding affinity (Km) of APEH-3*_Ss_* and APEH*_Ss_ versus* Ac-L-*p*NA, the typical archaeal substrate of this protease family, but the largest discrepancy was shown in the Km values for Ac-A-*p*NA, the most common substrate of mammalian APEHs, with the Km of APEH*_Ss_* at least 10-fold higher than that measured for APEH-3*_Ss_*. These data showed that the replacement of a Leu with an Ala residue did not affect the affinity of APEH-3*_Ss_* for the substrate. These observations were emphasized when looking at the catalytic efficiencies (kcat/Km) (**Table 2**) of all the archaeal APEH towards the two acetyl-amino acids. On the other hand, when the Ac-F-*p*NA was used as substrate, the kcat/Km values of both APEH-3*_Ss_* and APEH*_Ss_* were in the same range, but much more lower than those measured for the other archaeal APEHs which recognized Phe as the preferred acetyl-amino acid. These results indicate that APEH-3*_Ss_* has broad substrate specificity, preferring hydrophobic amino acids with small and large side chains. However, due to the different specificities of the two *S. solfataricus* proteases, a high structural identity of the substrate binding sites cannot be expected, likely the S1 subsite of APEH-3*_Ss_* could undergo conformational changes, indicative of the plasticity of the binding site.

**Table 2:** Kinetics parameters of APEH-3*_Ss_* compared with those of APEH from archaeal and mammalian sources.

Substrate	Enzyme*	Km	kcat	kcat/Km	*Ref.*
		(mM)	(sec^−1^)	(sec^−1^ mM^−1^)	
**Exopeptidase activity**					
	**APEH-3** ***_Ss_***	**0.6±0.05**	**18.0±0.1**	**32.7±0.4**	***This work***
	APEH*_Ss_*	0.3	5.0	20.0	[***9***]
aAc-L-*p*NA	APEH*_Ph0594_*	n.d.	n.d.	82.0	[***8***]
	APEH*_Ph0863_*	11	330	30.0	[***7***]
	APEH*_Ap1547.1_*	0.4	9.3	20.0	[***47***]
	**APEH-3** ***_Ss_***	**0.8±0.06**	**14.0±0.1**	**17.5±0.1**	***This work***
	APEH*_Ss_*	11.4	5.0	0.4	[***9***]
Ac-A-*p*NA	APEH*_Ph0594_*	n.d.	n.d.	0.3	[***8***]
	APEH*_Ph0863_*	18.4	17.3	0.9	[***7***]
	APEH*_Ap1547.1_*	n.d.	n.d.	0.5	[***6***]
	APEH*_S.scrofa_*	1.8	4.0×10^3^	2.3×10^3^	[***6***], [***11***]
	**APEH-3** ***_Ss_***	**4.0±0.2×10^−2^**	**8.0±0.5×10^−2^**	**2.0±0.01**	***This work***
Ac-F-*p*NA	APEH*_Ss_*	1.4×10^−1^	8.0×10^−1^	5.7	[***9***]
	APEH*_Ph0594_*	n.d.	n.d.	1.0×10^3^	[***8***]
	APEH*_Ap1547.1_*	n.d.	n.d.	7.6×10^2^	[***23***]
**Endopeptidase activity**					
bSuc-GGF-*p*NA	**APEH-3** ***_Ss_***			**1.3±0.1**	***This work***
	APEH*_Ss_*			2.1±0.2	[***9***]
c *Z*-GGL-*p*NA	**APEH-3** ***_Ss_***			**0.7±0.05**	***This work***
	APEH*_Ss_*			3.3±0.5	***This work***
Suc-AAA-*p*NA	**APEH-3** ***_Ss_***			**0.2±0.01**	***This work***
	APEH*_Ss_*			n.a.	***This work***
Suc-AAPF-*p*NA	**APEH-3** ***_Ss_***			**n.a.**	***This work***
	APEH*_Ss_*			1.0±0.1	***This work***
Suc-AAVA-*p*NA	**APEH-3** ***_Ss_***			**n.a.**	***This work***
	APEH*_Ss_*			n.a.	***This work***

* The Uniprot accession numbers are the following: Q7LX61 for APEH*_Ss_*; Q97VD6 for APEH-3*_Ss_*; Q9YBQ2 for APEH*_Ap1547.1_*; O58593 for APEH*_Ph0863_*; O58323 for APEH*_Ph0594_*; P19205 for APEH*_S.scrofa_*.

a Ac = Acetyl;

b Suc = Succinyl;

c Z = benzyloxycarbonyl.

In a previous study, it was reported that APEH*_Ss_* [9], which was regarded as an exopeptidase, exhibited endopeptidase activity like the other hyperthermophilic APEHs so far characterized. This underlines that, while archaeal APEHs were less specialized displaying endo- and exopeptidase activities, the mammalian APEHs are ‘true’ exopeptidases, as correctly classified.

As shown in **Table 2**, APEH*_Ss_* and APEH-3*_Ss_* varied drastically in their endopeptidase activity when peptide substrates differing in size and nature of amino acids were tested. Specifically, while APEH-3*_Ss_* was more effective in cleaving three-peptide substrates following an F residue, APEH*_Ss_* preferred an L residue ,and was unable to hydrolyze the three-peptide succinyl-alanine-alanine-alanine-para-nitroanilide (Suc-AAA-*p*NA). In addition, none of the analyzed tetra-peptides were recognized as substrates by APEH-3*_Ss_* whereas APEH*_Ss_* was active *versus* succinyl-alanine-alanine-proline-phenylalanine-para-nitroanilide (Suc-AAPF-*p*NA). These data suggest that APEH-3*_Ss_* is an exopeptidase with a pseudo-endopeptidase activity, due to the ability to hydrolyze only oligopeptides with a maximum length of three amino acids. To support this hypothesis, hydrolysis experiments were carried out on the nona-peptide bradykinin followed by HPLC and Edman degradation analyses. Results clearly demonstrated that while APEH*_Ss_* showed endopeptidase activity with a cleavage specificity after F or G residues (**Fig. 2E**), by contrast APEH-3*_Ss_* did not result in any peptide fragments in the reaction mixture.

### APEH-3*_Ss_* inhibitory activity of SsCEI

Protease inhibitors traditionally rely upon warhead motifs for tight binding to the desired target. We previously identified the first protein able to efficiently inhibit APEH from both *S. solfataricus* and mammalian sources, including the human isoform, with similar IC_50_ values in the nanomolar range. This inhibitor, named SsCEI (*Sulfolobus solfataricus* chymotrypsin-elastase inhibitor) [25], acted as a typical competitive inhibitor against APEH [20]. In order to obtain a better understanding of the physiological role of SsCEI, inhibition analyses were performed by pre-incubating APEH-3*_Ss_* with increasing amounts of the inhibitor and the half maximal (50%) inhibitory concentration (IC) of SsCEI calculated. The calibration curve for SsCEI followed a hyperbolic pattern and IC_50_ values were calculated as 0.8±0.05×10^−2^ µM or 2.0±0.1×10^−2^ µM using Ac-L-*p*NA or succinyl-glycine-glycine-phenylalanine-para-nitroanilide (Suc-GGF-*p*NA), respectively. Therefore, although the sequence identity between the catalytic domains of APEH-3*_Ss_* and APEH*_Ss_* is quite low (28%), SsCEI was able to specifically inhibit APEH-3*_Ss_*, possibly due to high flexibility of the protease structure, which is an important factor in the effective inhibition mechanism. These results suggest that SsCEI might play a role in fine-tuning these proteases, implicated in diverse physiological processes during *S. solfataricus* development. Although regulation of protease activity by endogenous inhibitors is well documented in Bacteria and Eukarya [26], [27], to date there are few reports on protease inhibitors in Archaea [25] and our findings indicate that the protease activity regulation by endogenous inhibitors is a conserved mechanism in the three domains of life.

### Secondary structural analysis of APEH-3*_Ss_*

**Figure 3:**
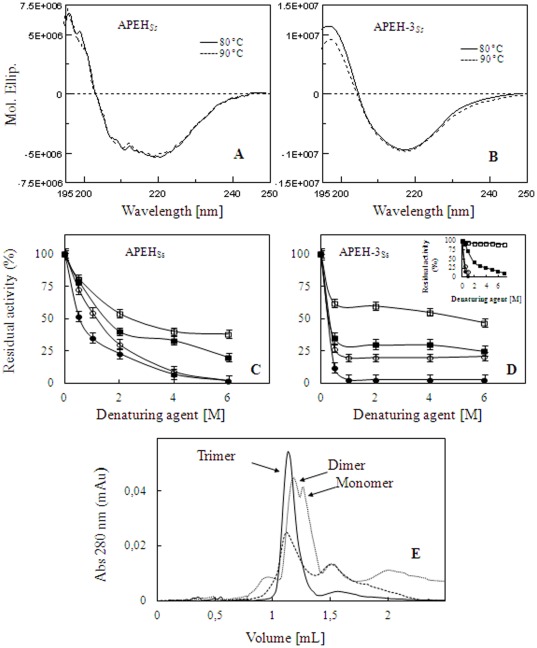
APEH structure and function analysis. (**A**) CD spectra performed at 80°C and 90°C for APEH*_Ss_* or (**B**) APEH-3*_Ss_*, respectively. (**C**) Effect of chaotropic agent on APEH*_Ss_*, and (**D**) APEH-3*_Ss_*: the enzymatic activity was measured using Ac-A-*p*NA for APEH-3*_Ss_* and Ac-L-*p*NA for APEH*_Ss_* as substrates, after addition of urea (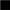) or guanidine hydrochloride (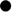); the activities were recovered after renaturation by extensive dialysis (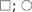); by comparison, mammalian APEH behavior [*ref* 28] is shown in **D** ***insert***. (**E**) Size exclusion chromatography elution profiles: native APEH-3*_Ss_* (**^__^**); APEH-3*_Ss_* treated with GnHCl (**•••**); GnHCl-treated APEH-3*_Ss_* after extensive dialysis (**---**).

To investigate the effect of temperature on the secondary structure of APEH*_Ss_* and APEH-3*_Ss_*, the far-UV CD spectra (195 to 250 nm wavelength range) of the two enzymes were recorded at 80°C and 90°C, which represent the *optimum* temperatures for APEH*_Ss_* and APEH-3*_Ss_* activities, respectively. CD measurements recorded at 80°C and 90°C showed that APEH*_Ss_* could adopt a possible intermediate conformation between α-helical (two minima at 208 nm and 222 nm and a typical maximum at 195 nm) and β-sheet (minimum at 218 nm and maximum at 195 nm) structure (**Fig. 3A**). However, the plots obtained at 80°C for APEH-3*_Ss_* showed a typical β-sheet structure with a single minimum peak at 218 nm and another maximum at 195 nm. As the temperature was increased to 90°C, the far-UV CD spectrum of APEH-3*_Ss_* remained essentially the same in terms of its overall features (**Fig. 3B**). Since molar ellipticity is an optical property of the protein molecules closely related to their conformational state, our results showed that by changing temperature, a very low or null loss in the ordered secondary structure of the two enzymes was observed. The invariance of CD spectra suggests that the two enzymes are structurally thermostable and that the different enzymatic activities of the two APEH are not due to thermally induced unfolding.

### Effect of chaotropic agents on the structure-function of acylpeptide hydrolase

Knowledge of the stability of proteins is essential to the understanding of their structures and functions. It is well established that proteins can be unfolded in aqueous solution by high concentrations of reagents such as guanidine hydrochloride (GnHCl) or urea. Denaturation with these chemicals is one of the primary ways of measuring the conformational stability of proteins. Previous studies on the porcine APEH have shown that this protease responds differently to treatment with urea (0.5–6.0 M) or GnHCl (0.5–4.0 M) (**Fig. 3D** ***insert***) [28].

Based on these studies, similar experiments were conducted on APEH*_Ss_* and APEH-3*_Ss_* in order to obtain information on the influence of these denaturants on archaeal enzymes. As shown in **Fig. 3C, D**, the enzyme activities of APEH*_Ss_* and APEH-3*_Ss_* decreased as the urea concentration increased following a different trend but reaching a similar residual activity of about 30% at 6 M urea [10]. However, both enzymes regained activity at almost all urea concentrations upon removal of the denaturing agent by dialysis and restoration of conditions favoring the native state, but this recovery was obtained to different extents, with the renaturating process being more efficient for APEH-3*_Ss_* (**Fig. 3C, D**). In addition, in the presence of 1 M GnHCl, the activity of APEH-3*_Ss_* was completely lost, as for APEH porcine, while 6 M GnHCl was needed to fully inactivate APEH*_Ss_*. However after removal of GnHCl only APEH-3*_Ss_* was able to recover about 25% activity, suggesting that the GnHCl-induced denaturation process is different from that of urea and irreversible in the case of APEH*_Ss_*.

As shown in **Fig. 3**, the trend of the unfolding/refolding pathways of APEH-3*_Ss_* was similar to that observed with the porcine APEH. Therefore we were interested in analyzing the changes in the oligomeric status of the enzyme APEH-3*_Ss_* in the presence of GnHCl. The alteration in the molecular size of the homotrimeric APEH-3*_Ss_* at 0.5 M GnHCl was investigated by gel exclusion chromatography. Native APEH-3*_Ss_* was eluted as trimeric enzyme with a molecular mass of 192 kDa (**Fig. 3E**) but after 0.5 M GnHCl-treatment, monomeric and dimeric species alone were revealed, with a molecular mass of 65 kDa and 110 kDa, respectively, both showing a low but detectable enzymatic activity. However, when the denatured enzyme was dialyzed and gel-filtered, the only trimeric form was again observed, but recovery of the enzymatic activity was incomplete (about 30%). This behavior could be due to the formation of no active intermediate species, as shown by the presence of additive peaks, which apparently did not correspond to any of the forms provided for APEH-3*_Ss_* (**Fig. 3E**). Furthermore, total subunit dissociation was also observed by treatment of APEH-3*_Ss_* or APEH_Ss_ with 1 M GnHCl, although following extensive dialysis about 20–25% of enzyme activity was restored, along with an incomplete reassociation into the native complexes (data not shown).

Therefore, it may be suggested that under GnHCl-induced denaturing conditions, the unfolding process follows a general stepwise dissociation mechanism which is common to archaeal and eukaryal APEHs. Conversely, the hyperthermophilic APEHs seem to be more efficient than the mammalian counterparts [28] in recovering their native-fold, possibly due to a high structural plasticity which allow the cells to survive in their natural extreme environments.

The unusual stability of APEHs from *S. solfataricus* could provide excellent models for studying the folding-unfolding behavior of the α/β hydrolase cluster of proteins.

### Tuning *apeh* gene expression in response to environmental stresses

Total RNA from *S. solfataricus* cells collected during growth under standard (80°C), oxidative (H_2_O_2_) or thermal stress conditions (85°C) was extracted and the expression levels of *sso1419*, *sso2141* (APEH*_Ss_*) and *sso2693* (APEH-3*_Ss_*) were examined by qRT-PCR. Unfortunately, under the culture conditions used, the levels of *sso1419* were possibly too low to perform a quantitative analysis, thus this gene was excluded from further investigation.

**Figure 4:**
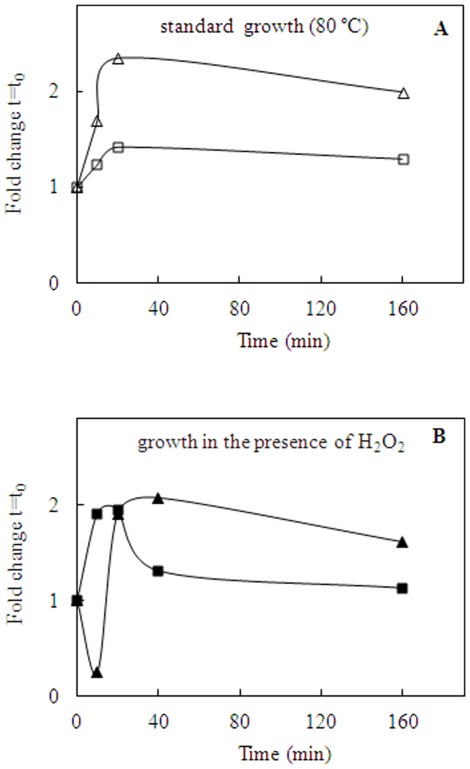
Transcriptional analysis under oxidative stress. (**A–B**) Comparisons of *apeh_Ss_* (squares) and *apeh-3_Ss_* (triangles) fold changes of gene transcriptions under standard **(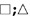)** or oxidative **(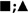)** conditions, normalized respect to the expression level at t = t_0_ of 23S gene.

**Figure 5:**
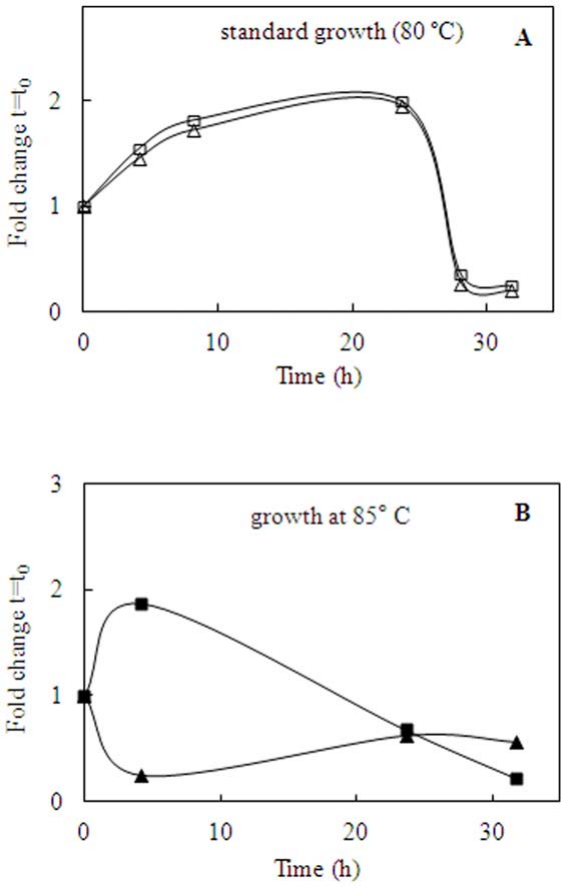
Transcriptional analysis under thermal stress. (**A–B**) Comparisons of *apeh_Ss_* (squares) and *apeh-3_Ss_* (triangles) fold changes of gene transcriptions under standard **(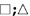)** or thermal stress **(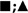)** conditions, normalized respect to the expression level at t = t_0_ of 23S gene.

The cell cultures in different experimental conditions were carried out in duplicate. All data shown are expressed as mean fold changes from triplicates, normalized against the expression level of the 23S gene (*ssor04*), the endogenous control, and calculated using the comparative Ct method [29]. In **Fig. 4A, B** and **Fig. 5A, B** the expressions levels of *apeh_Ss_* and *apeh-3_Ss_* genes were calculated with respect to the reference gene at t = t_0_, whereas in **Figure S2A, B** they were calculated with respect to the reference gene at each time of growth.

Under oxidative stress conditions, the growth rate was slow and the doubling time was around 11–13 h compared to that of 7–9 h observed in standard (80°C) cell cultures (data not shown). The effect of H_2_O_2_ addition on gene expression was detected at very short times, possibly due to the H_2_O_2_-degradation rate, at high temperatures. Specifically, under standard conditions, the expression level of *apeh_Ss_* was lower than that of *apeh-3_Ss_* (maximum fold change, at 20 min, is 1.41 *vs.* 2.34, respectively; **Fig. 4A**), but H_2_O_2_ supplementation caused up-regulation of the former and a concomitant down-regulation of the latter (**Fig. 4B**). Transcripts turned to standard condition levels when the effects of H_2_O_2_ vanished, as little as 20 minutes after its addition. Interestingly the relative expression levels of the two genes at each time of growth, as shown in **Figure S2A**, indicated an increase in *apeh_Ss_* expression in comparison to *apeh-3_Ss_* during the exponential growth phase, which was less marked under standard conditions (**Figure S2A** ***insert***).

Furthermore, when the cells were cultured at 85°C, the doubling time (12 h) increased to about 4 h with respect to that measured under standard conditions (80°C). The analysis conducted during the first 30 h of growth showed transcriptional patterns which were basically identical for *apeh_Ss_* and *apeh-3_Ss_* under standard conditions (**Fig. 5A**). However, thermal stress induced an up-regulation of *apeh_Ss_* during the early phase of growth (up to 4 hours) followed by a considerable decrease in transcript levels at longer times. Interestingly, the same analysis showed specular changes in *apeh-3_Ss_* gene expression (**Fig. 5B**). When the relative expression levels of the two genes were compared at each time point (**Figure S2B**), a different outline was obtained confirming a higher expression level for *apeh_Ss_* during the exponential growth phase, more evident in the standard conditions (**Figure S2B** ***insert***).

Therefore, under oxidative or thermal stress conditions the two *apeh* genes were differently regulated. Specifically, *S. solfataricus* cells responded to stress by over-expressing the *apeh_Ss_* and down-regulating the *apeh-3_Ss_*. It appears that this regulation involves these two genes in a complementary manner, so that when *apeh_Ss_* expression is triggered, as an immediate response to stress, *apeh-3_Ss_* is down-regulated. This trend could be related to the different properties of the two enzymes which apparently have complementary functions.

### Comparative analysis of the archaeal *apeh*-genes

Starting from the phylogenetic pathway of the S9 family (clan SC) described by Krem and Di Cera [30]–[32], based on sequence changes in and around the highly conserved amino acids of the catalytic domain, we tried to predict the place of the five archaeal APEHs characterized to date (**Figure S3**). Interestingly, by applying the model proposed by Krem and Di Cera, APEH-*3_Ss_* seemed to share an evolutionary lineage with an APEH from *Pyrococcus horikoshy* (APEH_Ph0863_) but not with the cognate enzyme from *S. solfataricus* (APEH*_Ss_*), which may follow a distinct branch. This was unexpected because isoenzymes from the same organisms usually belong to the same lineage. However, the lack of detailed biochemical studies on APEH from diverse sources makes reconstruction of the evolutionary history of this protease family difficult.

**Figure 6:**
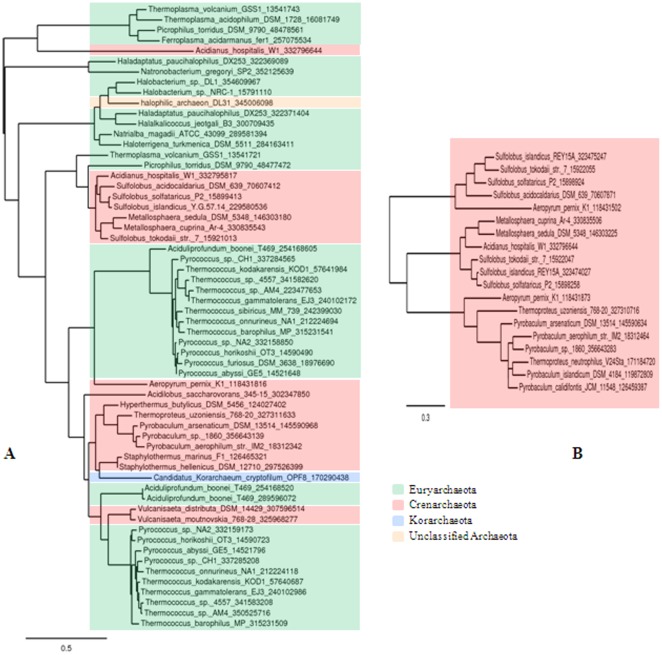
Phylogenetic analysis of APEH*_Ss_* and APEH-3*_Ss_* proteins in the archaeal kingdom. The cladogram shown in **A** (APEH*_Ss_*) includes 43 archaeal organisms, while in the tree shown in **B** (APEH-3*_Ss_*) only 15 species were retrieved, all belonging to *Crenarchaeota phylum*. The organisms in both trees are color coded according to the NCBI taxonomy. The trees were constructed by running Blast Explorer [41], in order to identify public similar sequences. For each run, proteins corresponding to hits pertaining to the Archaea, with BLAST e-value lower than 1E-20 and covering at least 50% of query sequence, were selected and fed to the “One Click Mode Phylogeny analysis” pipeline available on www.phylogeny.fr.

In addition, we investigated the distribution of APEH*_Ss_* and APEH-3*_Ss_* proteins in the entire archaeal kingdom. Cladograms reported in Fig. 6, were obtained by a user friendly web service (http://www.phylogeny.fr/), dedicated to reconstructing and analyzing phylogenetic relationships between molecular sequences through connection of several programs recognized for both speed and accuracy (see Methods). Following this approach, putative APEH*_Ss_* – homologues were found to be spread across three major *phyla* of the archaeal domain (*Euryarchaeota*, *Crenarchaeota*, *Korarchaeota*) and in one unclassified archaeal specie (**Fig. 6A**). By contrast, the same analysis predicted APEH-3*_Ss_* homologs to be both less abundant and restricted to the *Crenarchaeota phylum* (**Fig. 6B**), suggesting that APEH-3*_Ss_* could represent the founder of a new archaeal sub-family of APEH showing structural/functional properties intermediate between those of the enzymes from eukaryal and archaeal kingdoms.

### Conclusions

Oxidative stress such as free radicals and other pro-oxidants cause continuous formation of damaged proteins within cells [17]. In mammalian cells, the prevention of accumulation of oxidized cellular proteins is one of the major functions of the degradatory machinery, which rapidly eliminates damaged proteins whose increasing amount would interfere with normal cell function and viability. The most critical enzyme responsible for degradation of misfolded proteins is the proteasome, but it has been recently demonstrated that APEH has a cooperative role in protein turnover, also conferring cellular resistance to pro-oxidative reagents such as H_2_O_2_ [17]. Moreover, APEH has been postulated to serve as a key regulator of N-terminally acetylated proteins, which represent more than 80% of the cytosolic proteins in human cells [13]. With this in mind, what about archaeal microorganisms, which unlike their mammalian counterparts, show a multiplicity of *apeh* genes whose function is still unknown?

In this study, we investigated for the first time the possible role of APEH in a hyperthermophilic microorganism, providing a good starting point for understanding the biological function of this class of enzymes in Archaea. To this aim, a comparative analysis of two APEHs isolated from *S. solfataricus* was carried out, investigating the structural/functional properties and the regulation of gene expression. Interestingly, results revealed that the different APEHs can be identified as stress-regulated proteins, possibly playing a complementary role in ensuring cell survival under diverse growth conditions. Specifically, differential gene expression patterns were observed in response to stresses, indicating that the dynamic environmental changes of the “extreme” native habitat could play a role in selecting from time to time the expression of the enzyme suitable for the cell's survival. Moreover we showed that, while APEH*_Ss_* displayed the typical properties of the archaeal APEHs so far characterized, the latest isolated APEH-3*_Ss_* could be representative of a new class of acylpeptide hydrolase, sharing more features with the mammalian counterparts and appearing only affiliated within the *Crenarchaeota phylum*.

## Materials and Methods

### Strain, media and standard growth conditions

*S. solfataricus* strain P2 (DSM 1617) standard growth was conducted aerobically at 80°C in 2-liter shaked flasks and in Brock's basal salt medium [33], containing 0.05% yeast extract (Bacto) and 0.2% sucrose supplemented with 0.2% tryptone (Bacto), buffered at pH 3.5 with sulphuric acid 10%. Growth of cells was checked by measuring the OD at 600 nm. The cultures were harvested at the late stationary phase (1.5 OD) and cells were recovered by centrifugation (2,000 g at 4°C for 20 min) and stored at −80°C until use.

### Culturing of *S. solfataricus* cells under stress conditions

Oxidative stress was created by adding H_2_O_2_ at a final concentration of 30 µM to *S. solfataricus* cultures in early exponential growth phase (0.2 OD_600_ nm). 40 ml aliquots were collected at different times (10, 20, 40, 160 min) from standard and H_2_O_2_-stressed cultures for RNA isolation.

*S. solfataricus* cell growth under heat stress conditions was performed at 85°C in the same medium used for standard growth (80°C). The cells were cultured following a similar procedure to that described above and 40 ml aliquots were removed from standard and heat stressed cultures at 4, 8, 24, 28, 32 h for RNA isolation.

All experimental data reported in this study relating to structural-functional analysis of the purified APEHs and *apeh* gene expression were carried out in triplicate on two different cell cultures.

### Enzyme purification

The enzyme purification was followed using Ac-L-*p*NA as substrate. The cell pellet from 0.5 L of *S. solfataricus* standard culture (1.5 OD_600_) was resuspended in TEK buffer (20 mM Tris-HCl, 0.1 mM EDTA, 120 mM KCl, pH 7.5) containing 0.1% Triton-100, using a resuspension ratio of 5 mL/g cell pellet, and the mixture was incubated for 30 min on ice. The cell lysate suspension was centrifuged at 15,000×g for 50 min at 4°C. The supernatant was dialyzed overnight against 25 mM Tris-HCl, pH 7.5, and adjusted to 100 mM NaCl by addition of solid salt (Buffer A). The sample was loaded onto a Q Sepharose Fast Flow column (0.8×30 cm) (Amersham Biosciences) pre-equilibrated in Buffer A using an AKTA_FPLC_ system (Amersham Biosciences). After an extensive wash of the column with the same buffer, bound proteins were eluted using a linear ionic strenght gradient from 100 to 500 mM NaCl in Buffer A at a flow rate of 1 mL·min^−1^.

The active fractions were pooled, dialyzed against 25 mM Tris-HCl, 0.5 M ammonium sulphate, pH 6.5 (Buffer A), and then applied to a Phenyl Sepharose column (1.6×2.5 cm) (Amersham Biosciences) connected to an AKTA_FPLC_ system (Amersham Biosciences) equilibrated with the same buffer. Bound proteins were eluted with a linear gradient (0–100%) of Tris-HCl 25 mM buffer, pH 6.5 (Buffer B), at a flow rate of 1 mL·min^−1^. The active fractions were pooled, dialyzed against 50 mM Tris-HCl pH 7.5 and then applied to a Mono Q column (5×50 mm) (Amersham Biosciences) connected to an AKTA_FPLC_ system (Amersham Biosciences) and equilibrated with the same buffer (buffer A). Proteins were eluted with a linear ionic gradient from 0 to 0.5 M NaCl in buffer A at a flow rate of 1 mL·min^−1^. The active fractions eluted were pooled and dialyzed against 50 mM sodium acetate pH 5 (Buffer A), and loaded onto a Mono S (1.6×50 mm) connected to a SMART System (Pharmacia). Bound proteins were eluted with a linear gradient from 0 to 0.5 M NaCl (0–100%) in the same buffer. Active fractions were pooled, dialyzed overnight against Tris-HCl 25 mM, pH 7.5, and the purified protease was stored at −20°C.

### Molecular mass determination

Protein homogeneity was estimated by SDS-PAGE [34] using 10% (w/v) acrylamide resolving gel. Standard proteins (Broad Range) were from New England BioLabs. Molecular mass of the native enzyme was determined using Superdex 200 HR 10/30 column (Pharmacia Biotech), calibrated with BioRad gel filtration standards (code 151-1901). Native molecular mass determination of purified protease was also performed by using a Superose 12 PC 3.2/30 column connected to the a SMART System and calibrated using the molecular mass standards described above. Protein concentration was determined according to Bradford assay method [35].

### Nano-HPLC-MS/MS analysis

Protein band of interest stained with Coomassie Brilliant Blue G250 was excised from a preparative (20×20×0.15 cm) SDS-PAGE (10%) and *in-gel* digestion was performed according to Shevchenko *et al.* [36]. Peptide mixture was analyzed by a quadrupole time of flight instrument (Q-Star Elite, Applied Biosystems) coupled online to a nano-HPLC system (Ultimate 3000, Dionex) following the procedure described in Gogliettino *et al.* [37]. Three independent nano-HPLC-ESI-MS/MS experiments were performed.

### N-terminal amino acid sequence analysis

The purified protease was subjected to automated Edman degradation, using a Perkin-Elmer Applied Biosystem 477A pulsed-liquid protein sequencer. The NCBI non redundant protein dataset was scanned with the PSI-BLAST program [38] using the experimental obtained N-terminal as query sequence. Multiple sequence alignments and identity scores were generated by the CLUSTALW program [39].

### Protease activity assays

The endopeptidase activity of the purified APEH-3*_Ss_* (SSO2693) was measured spectrophotometrically following the release of p-nitroanilide (*p*NA) from the chromogenic substrates: Suc-GGF-*p*NA, Z-GGL-*p*NA, Suc-AAA-*p*NA, Suc-AAPF-*p*NA, and Suc-AAVA-*p*NA, all from Bachem. The reaction mixture (1 mL), containing the appropriate amount of enzyme in 25 mM Tris-HCl buffer pH 7.5, was preincubated at 90°C for 2 min. Then, Suc-GGF-*p*NA was added and the release of *p*-nitroanilide (ε_410_ = 8800 M^−1^•cm^−1^) was measured by recording the absorbance increase at 410 nm on a Cary 100 SCAN (VARIAN) UV/Vis spectrophotometer, equipped with a thermostated cuvette compartment. Calculated activities were based on the initial linear phase of release. One unit of APEH activity (U) is defined as the amount of enzyme required to hydrolyze 1 µmol of substrate per min under the assay conditions. The aminopeptidase activity of APEH-3*_Ss_* was measured by using Ac-amino acid-*p*NA, such as Ac-L-*p*NA (Sigma), Ac-A-*p*NA (Bachem) and Ac-F-*p*NA (Sigma), following the standard assay procedure described above. Unless otherwise reported, Suc-GGF-*p*NA (0.25 mM) or Ac-L-*p*NA (0.2 mM) was the substrate used to measure the protease activity.

### Temperature and pH influence on APEH-3*_Ss_* activity

Determinations of temperature and pH optima were performed using Suc-GGP-*p*NA as substrate. Effect of pH was determined between pH 4.5 and 9.0 under the assay conditions described above. Citrate/phosphate buffer (50 mM) was used for pH values ranging from 4.5 to 6.5. For pH values between 7.5 and 9.0, citrate/phosphate buffer was replaced by Tris-HCl (50 mM) buffer. Relative activity was expressed as a percentage of the maximum of the enzyme activity under the standard assay conditions. The optimum temperature of the purified enzyme was determined using the standard assay conditions in the temperature range 37–95°C. The thermal stability was measured at 70°C and 90°C.

### Effect of chaotropic agents on APEH-3*_Ss_* activity

The purified enzyme was preincubated at 90°C with urea (from 0 to 8 M) or guanidine hydrochloride (from 1 to 4 M) in 0.05 M Tris-HCl buffer, pH 7.5. After 1 h, the incubation samples were directly assayed for residual activity (compared with that of the controls). In another set of experiments, the denaturant-treated enzyme was extensively dialyzed against 0.05 M Tris-HCl, pH 7.5, to completely remove the denaturants prior to assay.

The oligomeric status of the enzyme in 0.5 M GnHCl was determined using the Superose 12 3.2/30 gel filtration column connected to a SMART System. The protein treated with 0.5 M GnHCl was loaded on the column equilibrated with Tris-HCl (0.05 M, pH 7.5) containing 0.5 M GnHCl. The calibration was performed as described above. Protein refolding was carried out by dialyzing the enzyme extensively against Tris-HCl (0.05 M, pH 7.5) before gel filtration chromatography. Active fractions were loaded onto SDS-PAGE 10% gel.

### Circular dichroism spectroscopy

Circular dichroism (CD) spectra were obtained with a Jasco J-715 spectropolarimeter with 400 µl of 5.0×10^−8^ M protein in 10 mM Tris-HCl (pH 7.5). Hellma quartz cells of a 0.1 cm path length were used for far-UV (195 to 250 nm). The temperature of the sample cell was regulated by a PTC-348 WI thermostat. The spectra were signal-averaged by adding three scans and baseline corrected by subtracting a buffer noise spectrum.

### Kinetic constant determination

The kinetic parameters of APEH-3*_Ss_* were determined at 90°C and all experiments were carried out in triplicate on two different protein preparations. The assays were performed using the chromogenic substrates and the procedures mentioned above. Data were fitted to the Michaelis-Menten equation by a nonlinear regression with the GraphPad Prism software.

### Serine protease inhibitory activity of SsCEI

The IC_50_ value of the APEH inhibitor (SsCEI) isolated from *S. solfataricus* [25] was determined towards APEH-3*_Ss_* using Ac-L-*p*NA (0.2 mM) and Suc-GGF-*p*NA (0.2 mM) as protease substrates. Protease (0.16×10^−5^ mM) and increasing concentrations of SsCEI (1.63×10^−3^–16.3×10^−3^ µM) were mixed and pre-incubated for 30 min at 80°C (the optimum temperature for SsCEI inhibitory activity) in Tris-HCl 50 mM, pH 7.5, before the addition of the enzyme substrate. The final volume of reaction was 1 mL. The residual enzymatic activity was determined using the assay procedure described above. The IC_50_ values of SsCEI were derived fitting the experimental data with the GraphPad Prism software, through a nonlinear curve-fitting method and using a simple binding isotherm equation {%I = %I_max_•[I]/(IC_50_+[I]}.

### Enzyme hydrolysis of bradykinin

Substrate specificity of the purified proteases in the cleavage of the peptide bradykinin (Sigma) was investigated. Reactions were carried out by incubating 0.7 µg of APEH-3*_Ss_* in the presence of bradykinin (10 nmol) at 90°C in 0.2 ml of 50 mM Tris-HCl pH 7.5 for 30 min. The samples were then analyzed by reverse-phase HPLC (BioLC; Dionex) on a μBondapak C18 column (3.9 by 300 mm; Waters) eluted with a linear gradient (0 to 95% acetonitrile in 0.1% TFA) at a flow rate of 1 ml/min. Control samples incubated in the absence of the enzyme or bradykinin were run in parallel. Edman degradation analysis was performed on each collected fractions. The same procedure was performed using APEH*_Ss_* and 80°C as incubation temperature.

### RNA isolation and quantitative real-time PCR analysis

Total cellular RNA was extracted from *S. solfataricus* cells, according to the SV Total RNA Isolation System (Promega) protocol, with an on column DNase I step. Total RNA concentrations were determined using a Qubit® Fluorometer (Invitrogen). RNAs were then reverse transcribed using the Transcriptor First Strand cDNA Synthesis Kit (Roche). A total of 100 ng of reverse-transcribed complementary DNA, and its dilution series to calculate the efficacy of primers, were amplified by quantitative real-time PCR (qRT-PCR) on an iCycler iQ™ (Bio-Rad) using 300 nM gene-specific primers, Maxima® SYBR Green/Fluorescein qPCR Master Mix (2×) (Fermentas) and the following PCR conditions: 1 cycle at 95°C for 10 min, and 40 cycles of 95°C for 15 sec, 60°C for 30 sec, and 72°C for 30 sec. The expression level of 23S RNA gene (*ssor04*) was used as an internal control for normalization. Raw cycle threshold values (Ct values) obtained for *apeh_Ss_* (*sso2141*) and *apeh-3_Ss_* (*sso2693*), the “target” genes, were compared to the Ct value obtained for 23S transcript levels (“ref” gene). The final graphical data were derived from the R = (E_target_)^ΔCt_target (control - sample)^/(E_ref_)^ΔCt_ref (control - sample)^ formula [29], where “control” cells were those grown under standard conditions, and “sample” cells were those grown under stress conditions. Universal Probe Library Assay Design Center (https://www.roche-applied-science.com/sis/rtpcr/upl/index.jsp?id=UP030000) was used for designing primers. The primer couples utilized were: *apeh_Ss_*, 5′-CAATTAGTGGCGCTACCAGAA-3′ and 5′-GACACTAAGGAGGAATGGGAAA-3′; *apeh-3_Ss_*, 5′-AGGGCATAGGAAAGGGCTAA-3′ and 5′-CCTTCTTCTGGTAGCATTATTTCG-3′; 23S, 5′-GCTAGCCCGAAAGGGTGT-3′ and 5′-GGCTTTCACCAACCACCA-3′.

### Phylogenetic analysis

The phylogenetic trees of amino acid sequences of APEH*_Ss_* and APEH-3*_Ss_*, of all archaeal domains were obtained by using the programs available at http://www.phylogeny.fr/ [40]. The APEH*_Ss_* (Uniprot accession number P95915) and APEH-3*_Ss_* (Uniprot accession number Q97VD6) protein sequences were compared each one with the NCBI non redundant protein dataset (version available at the time 2012-01-06) by running Blast Explorer [41], in order to identify public similar sequences. The program was run with the default parameters. For each run, proteins corresponding to hits retaining to the archaeal domain, with BLAST e-value lower than 1*E-20 and covering at least 50% of query sequence, were selected and fed to the “One Click Mode Phylogeny analysis” pipeline available on Phylogeny.fr. This simple to use web service, is dedicated to reconstructing and analyzing phylogenetic relationships between molecular sequences [40], [41] and the “One Click Mode” is a pipeline already set up to run and connect programs recognized for both accuracy and speed to rebuild a robust phylogenetic tree from a set of sequences: MUSCLE for multiple alignment [42]; Gblocks for alignment curation [43]; PhyML for phylogeny [44]; TreeDyn for tree drawing [45]. In particular, PhyML is a Maximum Likelihood distance method that implements the fast approximate likelihood ratio test (aLRT) for branches [46], a useful complement to the (time-consuming) bootstrap analysis [45]. At the end of the procedure two different trees were obtained, composed of the predicted APEH*_Ss_* and APEH-3*_Ss_* homologous proteins respectively.

## Supporting Information

Figure S1 **Amino acid sequences alignment of C-terminal protein region of APEH-3_Ss_; APEH** ***_Ss_*** **; APEH** ***_Ap1547.1_*** **; APEH** ***_Ph0594_*** **; APEH** ***_Ph0863_*** **and APEH** ***_S.scrofa_*** **.** Significant residues have been boxed in colour: red boxed letters for catalytic triad; oxyanion binding pocket is green box enclosed. The color of the amino acid residues indicate the percentage identity such as imposed by the ClustalW software. The Uniprot accession numbers are the following: Q97VD6 for APEH-3*_Ss_*; Q7LX61 for APEH*_Ss_*; Q9YBQ2 for APEH*_Ap1547.1_*; O58323 for APEH*_Ph0594_*; O58593 for APEH*_Ph0863_*; P 19205 for APEH*_S.scrofa_*.
(PDF)

Figure S2 **Transcriptional analysis under stress conditions.** (**A**) Transcriptional levels of *apeh_Ss_* (white bars) with respect to *apeh-3_Ss_* (gray bars) genes under oxidative stress or standard (insert) conditions during the exponential growth phase. (**B**) Transcriptional levels of *apeh_Ss_* (white bars) with respect to *apeh-3_Ss_* (gray bars) genes under thermal stress or standard (insert) conditions during the exponential growth phase.
(PDF)

Figure S3 **APEHs phylogenetic categories based on evolutionary pathways as reproduced from Krem and Di Cera 2001** [**30**] **.** The table on the top of the figure summarizes the sequence motifs surrounding the evolutionary markers and the active site residues for the subfamilies S9 of clan SC used for the construction of the APEHs evolutionary pathway. In the table, conserved and active site residues (*) are in bold; the sequence variations are indicated. In the scheme, more likely evolutionary transitions are indicated by arrows; the dashed line indicates a less likely transition. The usage codons of the evolutionary pathway of APEH*_Ss_* (SSO2141) and APEH-3*_Ss_* (SSO2693) are gray-boxed.
(PDF)

Table S1 **Mass spectrometry analysis of** ***sso2693*** **gene product.** The 64 kDa band was excised from the PAGE and *in situ* digested with trypsin. The peptide sequences obtained by the LC-MS/MS analysis were used for the protein identification in *S. solfataricus* genome database.
(PDF)

Table S2 **Sequence identities among pairs of APEH proteins, after domain alignments performed with ClustalW.** The approximate length (in amino acids) of the protein region along which the alignment is performed is indicated in the “aa length” column. ClustalW scores were reported. The superposition is performed on a whole protein, or the catalytic domains. Uniprot accession numbers are the following: Q7LX61 for APEH*_Ss_*; Q97VD6 for APEH-3*_Ss_*; Q9YBQ2 for APEH*_Ap1547.1_*; O58593 for APEH*_Ph0863_*; O58323 for APEH*_Ph0594_*; P19205 for APEH*_S.scrofa_*.
(PDF)
