# Supplementary material for: Identification and Characterisation of a Novel Acylpeptide Hydrolase from Sulfolobus Solfataricus: Structural and Functional Insights
Source: PLoS One. 2012 May 24;7(5):e37921. doi: 10.1371/journal.pone.0037921 (PMC3360023; doi:10.1371/journal.pone.0037921)
Supplement: Figure S1 — Amino acid sequences alignment of C-terminal protein region of APEH-3Ss; APEH Ss ; APEH Ap1547.1 ; APEH Ph0594 ; APEH Ph0863 and APEH S.scrofa . Significant residues have been boxed in colour: red boxed letters for catalytic triad; oxyanion binding pocket is green box enclosed. The color of the amino acid residues indicate the percentage identity such as imposed by the ClustalW software. The Uniprot accession numbers are the following: Q97VD6 for APEH-3Ss; Q7LX61 for APEHSs; Q9YBQ2 for APEHAp1547.1; O58323 for APEHPh0594; O58593 for APEHPh0863; P 19205 for APEHS.scrofa. (PDF) [file pone.0037921.s001.pdf]

Figure S1

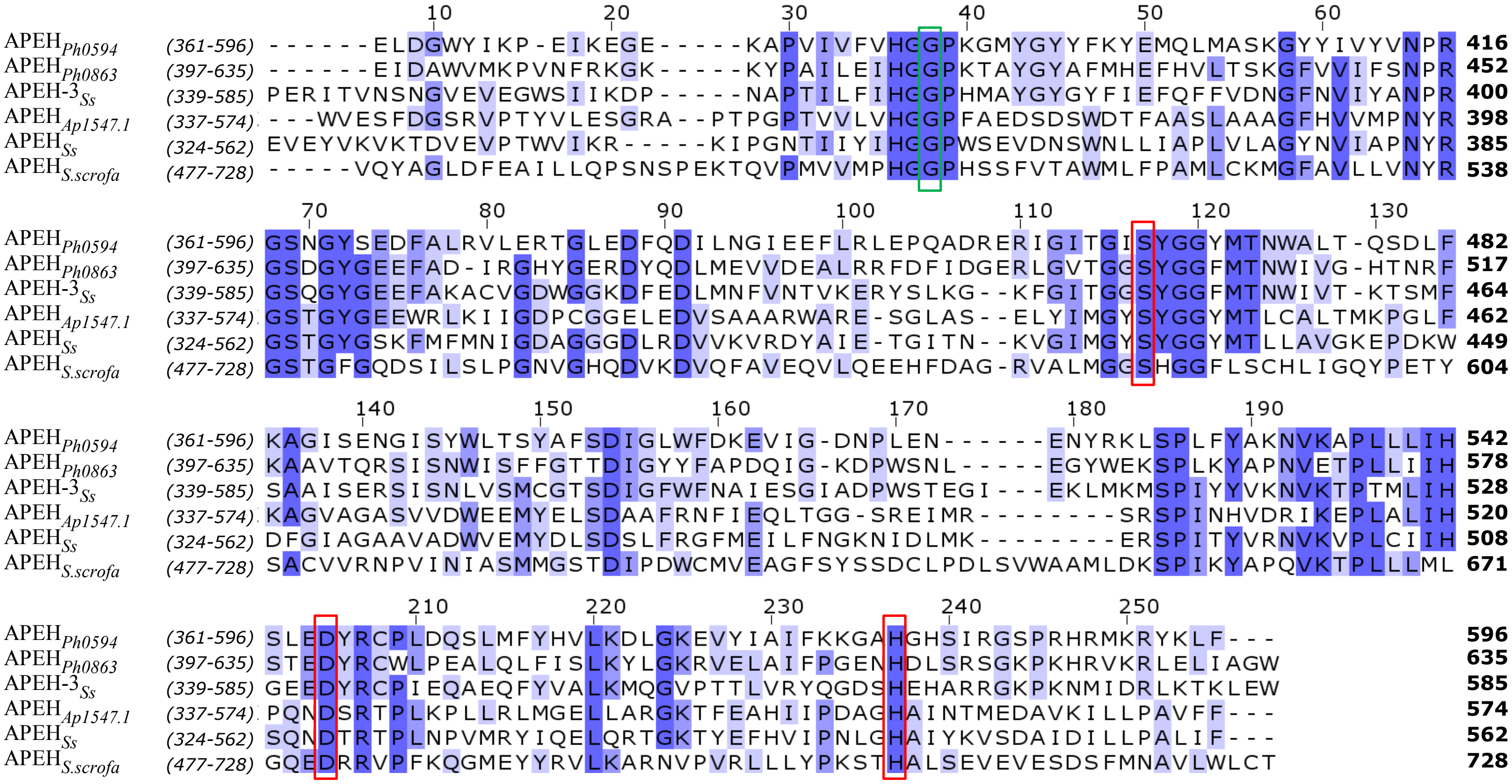
10 20 30 40 50 60
$APEH_{Ph0594}$ (361-596) ------ELDGWYIKP-EIKEGE-----KAPVIVFVHGGPKGMYGYYFKYEMQLMASKGYYIVYVNPR 416
$APEH_{Ph0863}$ (397-635) ------EIDAWVMKPVNFRKGK-----KYPAILEIHGGPKTAYGYAFMHEFHVLTSKGFVVIFSNPR 452
$APEH\text{-}3_{Ss}$ (339-585) PERITVNSNGVEVEGWSIIKDP-----NAPTILFIHGGPHMAYGYGYFIEFQFFVDNGFNVIYANPR 400
$APEH_{Ap1547.1}$ (337-574) ---WVESFDGSRVPTYVLESGRA--PTPGPTVVLVHGGPFAEDSDSWDTFAASLAAAGFHVVMPNYR 398
$APEH_{Ss}$ (324-562) EVEYVKVKTDVEVPTWVIKR-----KIPGNTIIYIHGGPWSEVDNSWNLLIAPLVLAGYNVIAPNYR 385
$APEH_{S.scrofa}$ (477-728) -----VQYAGLDFEAILLQPSNSPEKTQVPMVVMPHGGPHSSFVTAWMLFPAMLCKMGFAVLLVNYR 538
70 80 90 100 110 120 130
$APEH_{Ph0594}$ (361-596) GSNGYSEDFALRVLERTGLEDFQDILNGIEEFLRLEPQADRERIGITGISYGGYMTNWALT-QSDLF 482
$APEH_{Ph0863}$ (397-635) GSDGYGEEFAD-IRGHYGERDYQDLMEVVDEALRRFDFIDGERLGVTGGSYGGFMTNWIVG-HTNRF 517
$APEH\text{-}3_{Ss}$ (339-585) GSQGYGEEFAKACVGDWGGKDFEDLMNFVNTVKERYSLKG--KFGITGGSYGGFMTNWIVT-KTSMF 464
$APEH_{Ap1547.1}$ (337-574) GSTGYGEEWRLKIIGDPCGGELEDVSAAARWARE-SGLAS--ELYIMGYSYGGYMTLCALTMKPGLF 462
$APEH_{Ss}$ (324-562) GSTGYGSKFMFMNIGDAGGGDLRDVVKVRDYAIE-TGITN--KVGIMGYSYGGYMTLLAVGKEPDKW 449
$APEH_{S.scrofa}$ (477-728) GSTGFGQDSILSLPGNVGHQDVKDVQFAVEQVLQEEHFDAG-RVALMGGSHGGFLSCHLIGQYPETY 604
140 150 160 170 180 190
$APEH_{Ph0594}$ (361-596) KAGISENGISYWLTSYAFSDIGLWFDKEVIG-DNPLEN------ENYRKLSPLFYAKNVKAPLLLIH 542
$APEH_{Ph0863}$ (397-635) KAAVTQRSISNWISFFGTTDIGYYFAPDQIG-KDPWSNL-----EGYWEKSPLKYAPNVETPLLIIH 578
$APEH\text{-}3_{Ss}$ (339-585) SAAISERSISNLVSMCGTSDIGFWFNAIESGIADPWSTEGI---EKLMKMSPIYYVKNVKTPTMLIH 528
$APEH_{Ap1547.1}$ (337-574) KAGVAGASVVDWEEMYELSDAAFRNFIEQLTGG-SREIMR--------SRSPINHVDRIKEPLALIH 520
$APEH_{Ss}$ (324-562) DFGIAGAAVADWVEMYDLSDSLFRGFMEILFNGKNIDLMK--------ERSPITYVRNVKVPLCIIH 508
$APEH_{S.scrofa}$ (477-728) SACVVRNPVINIASMMGSTDIPDWCMVEAGFSYSSDCLPDLSVWAAMLDKSPIKYAPQVKTPLLLML 671
210 220 230 240 250
$APEH_{Ph0594}$ (361-596) SLEDYRCPLDQSLMFYHVLKDLGKEVYIAIFKKGAHGHSIRGSPRHRMKRYKLF--- 596
$APEH_{Ph0863}$ (397-635) STEDYRCWLPEALQLFISLKYLGKRVELAIFPGENHDLSRSGKPKHRVKRLELIAGW 635
$APEH\text{-}3_{Ss}$ (339-585) GEEDYRCPIEQAEQFYVALKMQGVPTTLVRYQGDSHEHARRGKPKNMIDRLKTKLEW 585
$APEH_{Ap1547.1}$ (337-574) PQNDSRTPLKPLLRLMGELLARGKTFEAHIIPDAGHAINTMEDAVKILLPAVFF--- 574
$APEH_{Ss}$ (324-562) SQNDTRTPLNPVMRYIQELQRTGKTYEFHVIPNLGHAIYKVSDAIDILLPALIF--- 562
$APEH_{S.scrofa}$ (477-728) GQEDRRVPFKQGMEYYRVLKARNVPVRLLLYPKSTHALSEVEVESDSFMNAVLWLCT 728
