# Supplementary figures and images for: Identification and Characterisation of a Novel Acylpeptide Hydrolase from Sulfolobus Solfataricus: Structural and Functional Insights
Source: PLoS One. 2012 May 24;7(5):e37921. doi: 10.1371/journal.pone.0037921 (PMC3360023; doi:10.1371/journal.pone.0037921)

Figure S2

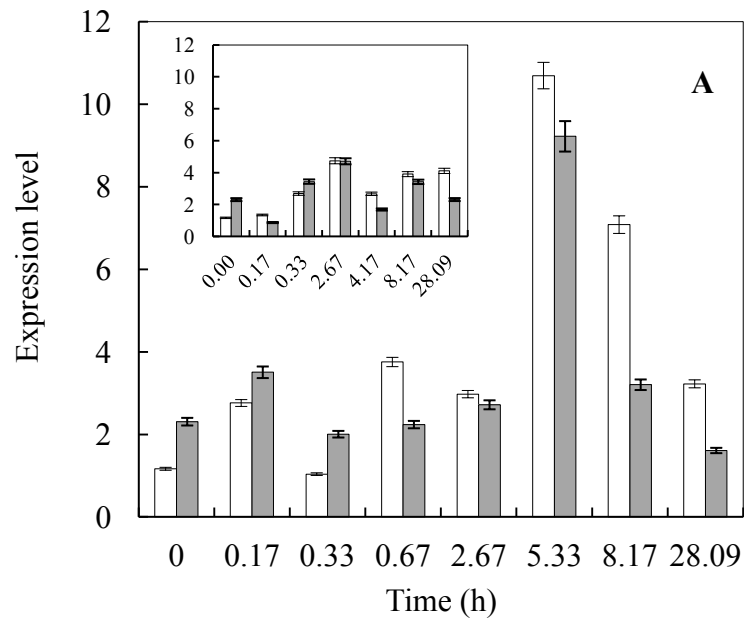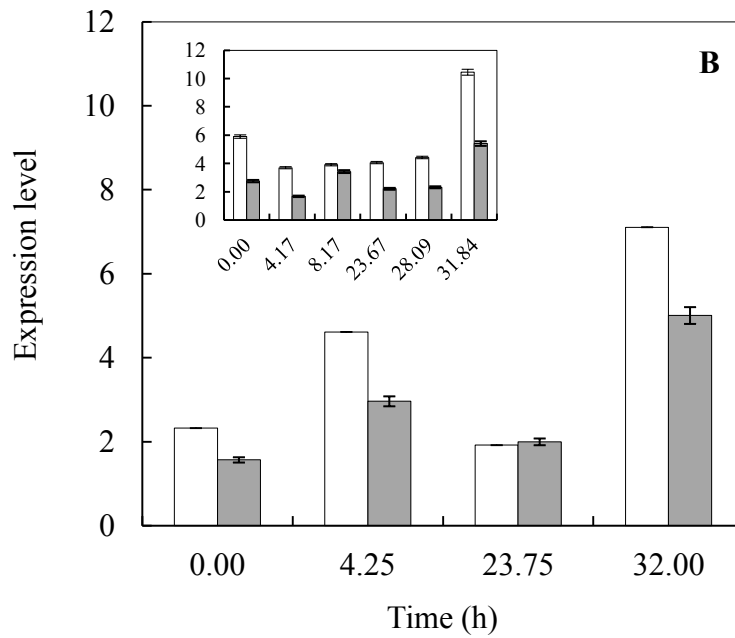

Supplement: Figure S2 — Transcriptional analysis under stress conditions. (A) Transcriptional levels of apehSs (white bars) with respect to apeh-3Ss (gray bars) genes under oxidative stress or standard (insert) conditions during the exponential growth phase. (B) Transcriptional levels of apehSs (white bars) with respect to apeh-3Ss (gray bars) genes under thermal stress or standard (insert) conditions during the exponential growth phase. (PDF) [file pone.0037921.s002.pdf]
