# Supplementary material for: Identification and Characterisation of a Novel Acylpeptide Hydrolase from Sulfolobus Solfataricus: Structural and Functional Insights
Source: PLoS One. 2012 May 24;7(5):e37921. doi: 10.1371/journal.pone.0037921 (PMC3360023; doi:10.1371/journal.pone.0037921)

Figure S3

X=S/G/M/T/A; **Y**= Y/V/D; **Z**= G/Y/I; **J**= G/S/P; **A**= E/N; **B**= G/N/D; **U**= G/N/S;
n= any residue.

A

**SC clan**	**Ser57**	**Ser146***	**Asp338* and Val/Cys341**	**His397***
**S9**	GGP*n***X***n*Y	GZ**S**YGG	J*n***AD***n*R and **T/C**	B*n*U**H**
SSO2693	GGPH**M**AY	GG**S**YGG ↑ **tcn**	GEE**D**YR **C**	GDS**H**
SSO2141	GGPW**S**EV ↑ tcn	GY**S**YGG ↑ **agy**	SQN**D**TR **T**	NLG**H**
PH0594	GGPK**G**MY	GI**S**YGG ↑ **agy**	SLE**D**YR **C**	GA-**H**
PH0863	GGPK**T**AY	GG**S**YGG ↑ tcn	STE**D**YR **C**	GEN**H**
APE1547.1	GGPF**A**ED	GY**S**YGG ↑ **agy**	PQN**D**SR **T**	DAG**H**

The ORFs correspond to: SSO2141 for $APEH_{Ss}$; SSO2693 for APEH-3_{Ss}; APE1547.1 for $APEH_{Ap1547.1}$; PH0863 for $APEH_{Ph0863}$; PH0594 for $APEH_{Ph0594}$

B

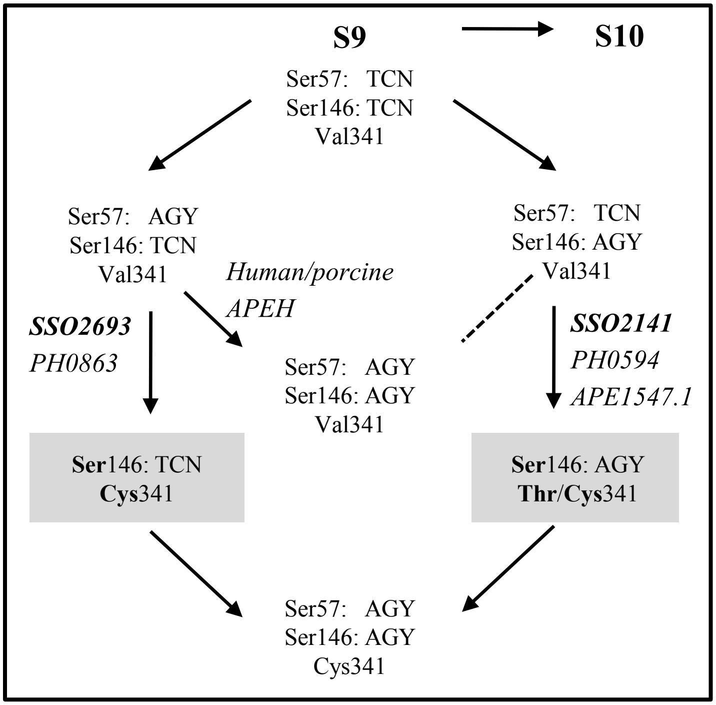

Supplement: Figure S3 — APEHs phylogenetic categories based on evolutionary pathways as reproduced from Krem and Di Cera 2001 [30] . The table on the top of the figure summarizes the sequence motifs surrounding the evolutionary markers and the active site residues for the subfamilies S9 of clan SC used for the construction of the APEHs evolutionary pathway. In the table, conserved and active site residues (*) are in bold; the sequence variations are indicated. In the scheme, more likely evolutionary transitions are indicated by arrows; the dashed line indicates a less likely transition. The usage codons of the evolutionary pathway of APEHSs (SSO2141) and APEH-3Ss (SSO2693) are gray-boxed. (PDF) [file pone.0037921.s003.pdf]
