# Supplementary material for: Identification and Characterisation of a Novel Acylpeptide Hydrolase from Sulfolobus Solfataricus: Structural and Functional Insights
Source: PLoS One. 2012 May 24;7(5):e37921. doi: 10.1371/journal.pone.0037921 (PMC3360023; doi:10.1371/journal.pone.0037921)
Supplement: Table S1 — Mass spectrometry analysis of sso2693 gene product. The 64 kDa band was excised from the PAGE and in situ digested with trypsin. The peptide sequences obtained by the LC-MS/MS analysis were used for the protein identification in S. solfataricus genome database. (PDF) [file pone.0037921.s004.pdf]

Table S1

Gel band (Mw)	ORF	m/z Observed	Mr Measured	Mr Calculated	Peptide	Peptide sequence
**64 kDa**	*sso2693*	606.87	1211.72	1211.68	12-22	**LVPEITIENGK**
		547.35	1092.69	1092.63	37-45	**SSIYLNLKR**
		717.89	1433.77	1433.74	72-84	**SSLLEAQLYGEPK**
		639.38	1915.13	1915.04	102-118	**GILVIAEENTDKTLPFR**
		602.99	1805.98	1805.89	149-165	**LVTGNFDVTDLATNGNR**
		636.03	1905.06	1904.95	195-212	**ITKEDGTVQAIAMNSEGK**
		689.40	1376.78	1376.73	249-260	**VLTDLFDGVKDR**
		488.79	975.57	975.52	291-299	**VTSGNIMVR**
		546.29	1635.86	1635.79	323-335	**YRDIEYDPNPNIK**
		586.79	1171.56	1171.51	401-411	**GSQGYGEEFAK**
		600.31	1198.61	1198.57	461-471	**TSMFSAAISER**
		500.79	999.56	999.51	511-518	**MSPIYYVK**
		551.33	1100.64	1100.60	549-558	**MQGVPTTLVR**
		445.28	888.54	888.49	574-580	**NMIDRLK**
