# Supplementary material for: Identification and Characterisation of a Novel Acylpeptide Hydrolase from Sulfolobus Solfataricus: Structural and Functional Insights
Source: PLoS One. 2012 May 24;7(5):e37921. doi: 10.1371/journal.pone.0037921 (PMC3360023; doi:10.1371/journal.pone.0037921)
Supplement: Table S2 — Sequence identities among pairs of APEH proteins, after domain alignments performed with ClustalW. The approximate length (in amino acids) of the protein region along which the alignment is performed is indicated in the “aa length” column. ClustalW scores were reported. The superposition is performed on a whole protein, or the catalytic domains. Uniprot accession numbers are the following: Q7LX61 for APEHSs; Q97VD6 for APEH-3Ss; Q9YBQ2 for APEHAp1547.1; O58593 for APEHPh0863; O58323 for APEHPh0594; P19205 for APEHS.scrofa. (PDF) [file pone.0037921.s005.pdf]

Table S2

Name	aa length Whole sequence	aa length Catalytic domain	Name	aa length Whole sequence	aa length Catalytic domain	Score Whole sequence	Score Catalytic domain
$APEH_{Ap1547.1}$	582	**238**	$APEH_{Ph0594}$	622	**235**	17.0	**26.0**
$APEH_{Ap1547.1}$	582	**238**	$APEH_{Ph0863}$	642	**239**	12.0	**20.0**
$APEH_{Ap1547.1}$	582	**238**	$APEH_{Ss}$	569	**239**	34.0	**48.0**
$APEH_{Ap1547.1}$	582	**238**	$APEH\text{-}3_{Ss}$	591	**247**	14.0	**23.0**
$APEH_{Ap1547.1}$	582	**238**	$APEH_{S.scrofa}$	732	**252**	18.0	**26.0**
$APEH_{Ph0594}$	622	**235**	$APEH_{Ph0863}$	642	**239**	28.0	**45.0**
$APEH_{Ph0594}$	622	**235**	$APEH_{Ss}$	569	**239**	17.0	**25.0**
$APEH_{Ph0594}$	622	**235**	$APEH\text{-}3_{Ss}$	591	**247**	21.0	**40.0**
$APEH_{Ph0594}$	622	**235**	$APEH_{S.scrofa}$	732	**252**	12.0	**22.0**
$APEH_{Ph0863}$	642	**239**	$APEH_{Ss}$	569	**239**	18.0	**23.0**
$APEH_{Ph0863}$	642	**239**	$APEH\text{-}3_{Ss}$	591	**247**	28.0	**44.0**
$APEH_{Ph0863}$	642	**239**	$APEH_{S.scrofa}$	732	**252**	15.0	**28.0**
$APEH_{Ss}$	569	**239**	$APEH\text{-}3_{Ss}$	591	**247**	15.0	**28.0**
$APEH_{Ss}$	569	**239**	$APEH_{S.scrofa}$	732	**252**	15.0	**25.0**
$APEH\text{-}3_{Ss}$	591	**247**	$APEH_{S.scrofa}$	732	**252**	13.0	**24.0**
